# JBP485, A Dual Inhibitor of Organic Anion Transporters (OATs) and Renal Dehydropeptidase-I (DHP-I), Protects Against Imipenem-Induced Nephrotoxicity

**DOI:** 10.3389/fphar.2022.938813

**Published:** 2022-06-08

**Authors:** Chong Wang, Changyuan Wang, Jingjing Wu, Qiang Meng, Huan Jin, Huijun Sun, Taiichi Kaku, Jing Chen, Xiaokui Huo, Kexin Liu

**Affiliations:** ^1^ Institute of Integrative Medicine, Dalian Medical University, Dalian, China; ^2^ Department of Clinical Pharmacology, College of Pharmacy, Dalian Medical University, Dalian, China; ^3^ Provincial Key Laboratory for Pharmacokinetics and Transport, Liaoning Dalian Medical University, Dalian, China; ^4^ Japan Bioproducts Industry Co. Ltd, Tokyo, Japan; ^5^ School of Chemistry and Materials Science, University of Science and Technology of China, Hefei, China; ^6^ Pharmaceutical Research Center, Second Affiliated Hospital, Dalian Medical University, Dalian, China

**Keywords:** Imipenem, JBP485, OATs, DHP-I, DDI

## Abstract

Imipenem (IMP) possesses a broad spectrum of antibacterial activity; however, nephrotoxicity limits its clinical application in patients with renal insufficiency. In our previous studies, a dipeptide, JBP485, a dipeptide with the chemical structure cyclo-trans-4-L-hydroxyprolyl-L-serine, was found to attenuate drug-induced kidney injury. The current study aimed to explore whether JBP485 could relieve IMP-induced kidney injury and clarify the potential molecular pharmacokinetic mechanism. The effects of JBP485 on IMP nephrotoxicity were evaluated in rabbits and human kidney 2 (HK-2) cells. Drug-drug interactions (DDIs) mediated by organic anion transporters (OATs) and dehydropeptidase-I (DHP-I) were explored through pharmacokinetic studies in rats, metabolism assays in the kidney, and uptake studies in OAT-over-expressing cells. The results revealed that JBP485 significantly ameliorated IMP-induced nephrotoxicity in rabbits. Further, incubation of HK-2 cells with JBP485 or cilastatin markedly improved the cell survival rate, inhibited apoptosis and attenuated mitochondrial damage by improving the stability of IMP and reducing its intracellular accumulation. This suggests that DHP-I and OATs might be involved in the protective effect of JBP485. Furthermore, coadministration with JBP485 significantly increased the IMP’s plasma concentration as well as the area under the plasma concentration-time curve (AUC), while decreasing IMP renal clearance and cumulative urinary excretion. Moreover, JBP485 reduced IMP uptake in kidney slices and OAT1/3-human embryonic kidney 293 (HEK293) cells. At the same time, the metabolism of IMP by DHP-I was inhibited by JBP485 with an IC_50_ value of 12.15 ± 1.22 μM. Finally, the molecular docking assay revealed a direct interaction between JBP485 and OAT1/3 or DHP-I. In conclusion, JBP485 protected against IMP nephrotoxicity in rabbits and HK-2 cells by improving IMP stability and reducing its intracellular accumulation via simultaneous inhibition of renal OATs and DHP-I. JBP485 is a promising renoprotective agent and could serve as an effective supplement to reduce IMP-induced adverse renal reactions in the clinical setting.

## Introduction

It has been proved that nephrotoxicity was a common side effect of several antibiotics. It not only limits the use of these drugs but can even cause severe kidney injury. Prevention of nephrotoxicity can increase patients’ drug tolerance, allowing higher doses of a drug to be administered for a longer period, thereby increasing the rate of successful treatment (Shayan and Elyasi, 2020). Imipenem (IMP), the first commercially available β-lactam agent of the carbapenem class, possesses broad-spectrum antibacterial activity *in vitro*, enclosing Gram-negative and Gram-positive aerobic and anaerobic species (Benfield and Chrisp, 1992). IMP is rapidly catalysed to toxic and inactive metabolites by the dehydropeptidase-I (DHP-I) enzyme at the luminal side of proximal tubular cells in the kidney; this leads to nephrotoxicity (Huo et al., 2019). Cilastatin is a specific inhibitor of DHP-I with no pharmacological effects; thus, it is usually used as a renoprotective agent in clinic. Therefore, IMP is formulated at a 1–1 mg ratio with cilastatin to prevent rapid hydrolysis of toxic metabolites with accumulation in tubular cells (Hakeam et al., 2019). In addition, our previous study found that both IMP and cilastatin are substrates of human organic anion transporter 1 (OAT1) and OAT3 (Huo et al., 2019; Zhu et al., 2020). Renal OATs have a central role in moving small-molecule endogenous metabolites, drugs and toxins (exogenous and endogenous) between tissues and interfacing body fluids (Nigam, 2018). Numerous evidence indicates that the residual renal secretory capacity, especially via OATs, may be particularly important in the setting of chronic kidney disease. In fact, it is known that the proximal tubule residual function is possible to be central to the remotion of compounds not commonly eliminated by haemodialysis, including many protein-bound small-molecule uremic solutes and toxins (Bush et al., 2020). In our previous studies, cilastatin was found to inhibit IMP transport by hOAT1/3, reduce hOAT1/3-dependent cytotoxicity, and alleviate nephrotoxicity induced by IMP in a concentration-dependent manner (Huo et al., 2019). Therefore, OATs and DHP-I could serve as targets to improve the therapeutic effect of IMP and decrease its toxicity.

JBP485 (cyclo-trans-4-L-hydroxyprolyl-L-serine), a dipeptide, was first isolated from Laennec (a trading name for the hydrolysate of the human placenta) (Cang et al., 2011). It displays notable anti-apoptosis and antioxidant properties (Wu et al., 2008; Yang et al., 2009; Cang et al., 2010; Liu et al., 2011; Wang et al., 2011). Our previous studies demonstrated that JBP485 regulates the expression of OATs and multi-drug resistance-associated protein 2/ABCC2 (MRP2) to attenuate drug-induced kidney injury (Liu et al., 2012; Guo et al., 2013). Moreover, several studies have demonstrated that JBP485 is a substrate of OATs and can inhibit the renal excretion of *p*-aminohippurate (PAH) (Zhang et al., 2010). Considering its OAT inhibitory activity and its antioxidant and anti-apoptosis properties, JBP485 may influence IMP nephrotoxicity when simultaneously administered.

Thus, the purpose of the current study was to explore whether JBP485 can relieve IMP-induced kidney injury and to clarify the mechanism underlying the drug-drug interaction (DDI) between IMP and JBP485. The results revealed that JBP485 ameliorated IMP-induced nephrotoxicity in rabbits and human kidney 2 (HK-2) cells. A liquid chromatography–tandem mass spectrometry (LC-MS/MS) method for determining IMP was established and an *in vivo* pharmacokinetic study, as well as an *in vitro* uptake assay of kidney slices and hOAT1/3-transfected cells, were performed to verify that JBP485 inhibited the renal excretion of IMP. At the same time, the metabolism of IMP by renal DHP-I was also found to be inhibited by JBP485. These findings indicate that OATs and DHP-I are the targets of the DDI between IMP and JBP485 and contribute to the protective effect of JBP485 against IMP-induced nephrotoxicity.

## Materials and Methods

### Materials

IMP was obtained from Dalian Meilun Biology Technology Co., Ltd. (Dalian, China). Cilastatin was purchased from Topscience Co., Ltd. (Shanghai, China). JBP485 was provided by Japan Bioproducts Industry Co., Ltd. (Tokyo, Japan). All other chemicals and reagents utilized in this study were of analytical purity grade and were commercially available.

### Animals

Male Wistar rats (weighing 220–250 g) and male New Zealand white rabbits (2.0–3.0 kg) were both obtained from the Experimental Animal Centre of Dalian Medical University (Dalian, China; permit number SCXK 2013-003). All animals were fed a chow diet and allowed free access to water. The animal experiments were executed based on the laboratory animals guidelines of the National Institutes of Health. The animals were fasted for 12 h before experiments, with access to water ad libitum.

### Cell Culture

Human embryonic kidney 293 (HEK293) cells and HK-2 cells were grown in Dulbecco’s modified Eagle’s medium (Invitrogen, Carlsbad, CA) and DMEM/F12 medium (KeyGen, Nanjing, China), respectively, supplemented with 10% (v/v) foetal bovine serum (Invitrogen), 100 U/ml penicillin and 100 mg/ml streptomycin. Cells were cultured at 37°C with a 5% (v/v) CO_2_ atmosphere and 95% relative humidity. Cell culture reagents were purchased from Gibco (Grand Island, NY).

### Biochemical Assay

The levels of blood urea nitrogen (BUN) and creatinine (CRE) were detected according to the instructions supplied by Nanjing Jiancheng Institute of Biotechnology (Nanjing, China).

### Toxicity Study in Rabbits

Rabbits were randomly divided into four groups: (1) control group, (2) IMP (200 mg/kg) group, (3) JBP485 (200 mg/kg) group, (4) IMP (200 mg/kg) + JBP485 (200 mg/kg) group. The doses chosen were set according to our previous study (Huo et al., 2019). Pre-treatment with JBP485 was performed by intraperitoneal administration one day in advance of the toxicity study. Test drugs were diluted in normal saline and were administered intravenously to rabbits via the ear vein at a rate of 5 ml/min. At 0, 24, 48 and 72 h after administration, blood samples (0.5 ml) were collected *via* the ear vein into heparin tubes and the samples were centrifuged at 1,000 × *g* for 10 min to obtain plasma. Plasma samples were stored at −20°C until analysis.

After 72 h, the rabbits were decapitated, and the kidneys should be immediately excised and fixed in neutral 10% buffered formalin. Histopathological examination was conducted through routine haematoxylin-eosin (HE) paraffin embedding. Tissue samples were sectioned and stained with HE.

### *In Vivo* Renal Clearance Experiments

Rats were randomly divided into two groups: 1) IMP alone (45 mg/kg) group, 2) IMP (45 mg/kg) + JBP485 (90 mg/kg) group. Test drugs were diluted in normal saline and were administered intravenously via the left jugular vein. After intravenous administration, blood samples (0.2 ml) were collected through the other side of the jugular vein with heparinized syringes at the time points: 1, 3, 5, 10, 30, 60, 120, 240, 360 and 480 min. After each blood sample collection, 0.2 ml isotonic saline solution was injected. Bladders were cannulated and urine was collected at 2, 4, 6 and 8 h after administration. LC-MS/MS method was used to measure IMP concentrations. Pharmacokinetic parameters, renal clearance (CL_R_) and cumulative urinary excretion were calculated.

### *In Vitro* Uptake in Kidney Slices

Rat kidneys were cut into slices with a ZQP-86 tissue slicer (Zhixin Co. Ltd., China), as previously described (Wang et al., 2014). After preincubation for 3 min at 37°C, the kidney slices were transferred to 24-well culture plates having 1 ml fresh oxygenated buffer with IMP (50 μM) and/or JBP485 (50 μM) for further incubation at 37°C under gentle shaking. After incubation for 5, 15 and 30 min, the kidney slices were washed using ice-cold Hanks’ balanced salt solution (pH 7.5). Accumulated IMP in the homogenized kidneys was determined by LC-MS/MS.

### Uptake By HK-2 Cells and OAT1/3-Transfected HEK293 Cells

HK-2 cells, hOAT1-HEK293 cells, hOAT3-HEK293 cells or mock cells were seeded in 24-well culture plates and cultured for 48 h before the experiment. The cells were washed twice. After adding transport buffer (1 ml) with IMP (50 μM) and/or JBP485 (50 μM), the uptake was initiated. The cells were incubated for 10 min in transport buffer at 37°C. Moreover, the concentration-dependent uptake of IMP and the effects of JBP485 on IMP uptake were examined. The cells were washed and lysed and then transferred into a polythene tube for quantization. Protein was measured according to the bicinchoninic acid procedure (Solarbio, Beijing, China) using bovine serum albumin for the standard.

### Metabolism of IMP By HK-2 Cells and Renal DHP-I

The stability of IMP in the medium of HK-2 cells was evaluated according to previously reported methods (Huo et al., 2019). Cells were seeded in 24-well culture plates and cultured for 48 h before each experiment. IMP (50 μM) was added into the medium with or without JBP485 (50 μM) or cilastatin (50 μM). After 0, 1, 2, 4 and 6 h, a 50 μl aliquot of the medium was sampled for determination of IMP by LC-MS/MS.

Renal DHP-I enzyme was obtained from rat renal tissue according to the methodology described in a previous study (Agudelo et al., 2014). Before treatment, DHP-I extract was equilibrated at 37°C for 0.5 h in the culture medium, in the absence or presence of JBP485 (50 μM). The reaction was initiated by the addition of the required concentrations of IMP. The reaction mixture (100 μl) was collected at 0.5, 1, 2 and 3 h to determine the remaining concentration of IMP. Moreover, the concentration-dependent metabolism of IMP (5-200 μM) and the effects of JBP485 (1-100 μM) on IMP (50 μM) metabolism were examined.

### Cytotoxicity Assay

The cytotoxicity of IMP on HK-2 cells was evaluated using CCK-8 assays. HK-2 cells were seeded in 96-well plates and cultured overnight. Fresh medium containing 0-5 mM IMP in the absence or presence of 500 μM JBP485 or 500 μM cilastatin was then added, and the cells were incubated for an additional 24 h. A CCK-8 assay (Solarbio, China) was used to determine cell viability.

### Cell Apoptosis Analysis

HK-2 cells were seeded in 6-well plates overnight and were cultured for 24 h in the presence of IMP (2 mM) with or without JBP485 (500 μM) or cilastatin (500 μM). HK-2 cells were then stained using an Annexin V-FITC Apoptosis Detection Kit (Beyotime Institute of Biotechnology, Shanghai, China) and analysed by flow cytometry.

### JC-1 Staining

HK-2 cells were seeded in 6-well plates and cultured overnight. Fresh medium containing IMP (2 mM) in the absence or presence of JBP485 (500 μM) or cilastatin (500 μM) was then added, and the cells were incubated for an additional 24 h. Then, the cells were stained with JC-1 (2 μM, Beyotime Institute of Biotechnology, Shanghai, China) and observed by a fluorescence microscope (Leica DM 14000B, Germany).

### Molecular Docking Simulation

The molecular docking simulation was conducted to explore the interactions between JBP485 and DHP-I, OAT1 and OAT3. The crystal structure homology models of DHP-I, OAT1 and OAT3 were built by Swiss-model (see “Supporting Information”). The Sybyl/Surflex module (RRID: SCR_000196) was used to simulate molecular docking. The Surflex-Dock program was used to generate the binding conformation of JBP485 to the three proteins using the default parameters. The total score of the binding results represents the affinities. Additionally, PyMOL Molecular Graphics System version 16.1.0.15350 (DeLano Scientific LLC) was used to visualize the docking results.

### LC-MS/MS Analysis

The concentrations of analysts in plasma, urine and cell lysate were quantitatively determined using an API 3200 LC-MS/MS system (Applied Biosystems, CA, United States). Chromatographic separation was performed on an Eclipse XDB-C8 column (150 mm × 4.6 mm, 5 μm; Agilent Technology Inc., CA, United States). Acetonitrile and water with 0.1% (v/v) formic acid were used as mobile phase. IMP and its internal standard (bestatin) were detected in positive mode with transitions of m/z 300.1 → 126.1 and m/z 309.1 → 120.3, respectively. PAH and ES were detected in negative mode with transitions of m/z 193.0 →149.0 and m/z 348.9→268, respectively.

### Data Analysis

Data are expressed as the mean ± standard deviation (SD). Statistical analysis was performed using SPSS 13.0 software. Student’s two-tailed *t*-tests were used when comparing two different groups. One-way ANOVA followed by Dunnett’s post hoc tests was used when comparing various groups. In all statistical analyses, *p* < 0.05 were considered statistically significant.

## Results

### Protective Effect of JBP485 on IMP-Induced Nephrotoxicity in Rabbits

**FIGURE 1:**
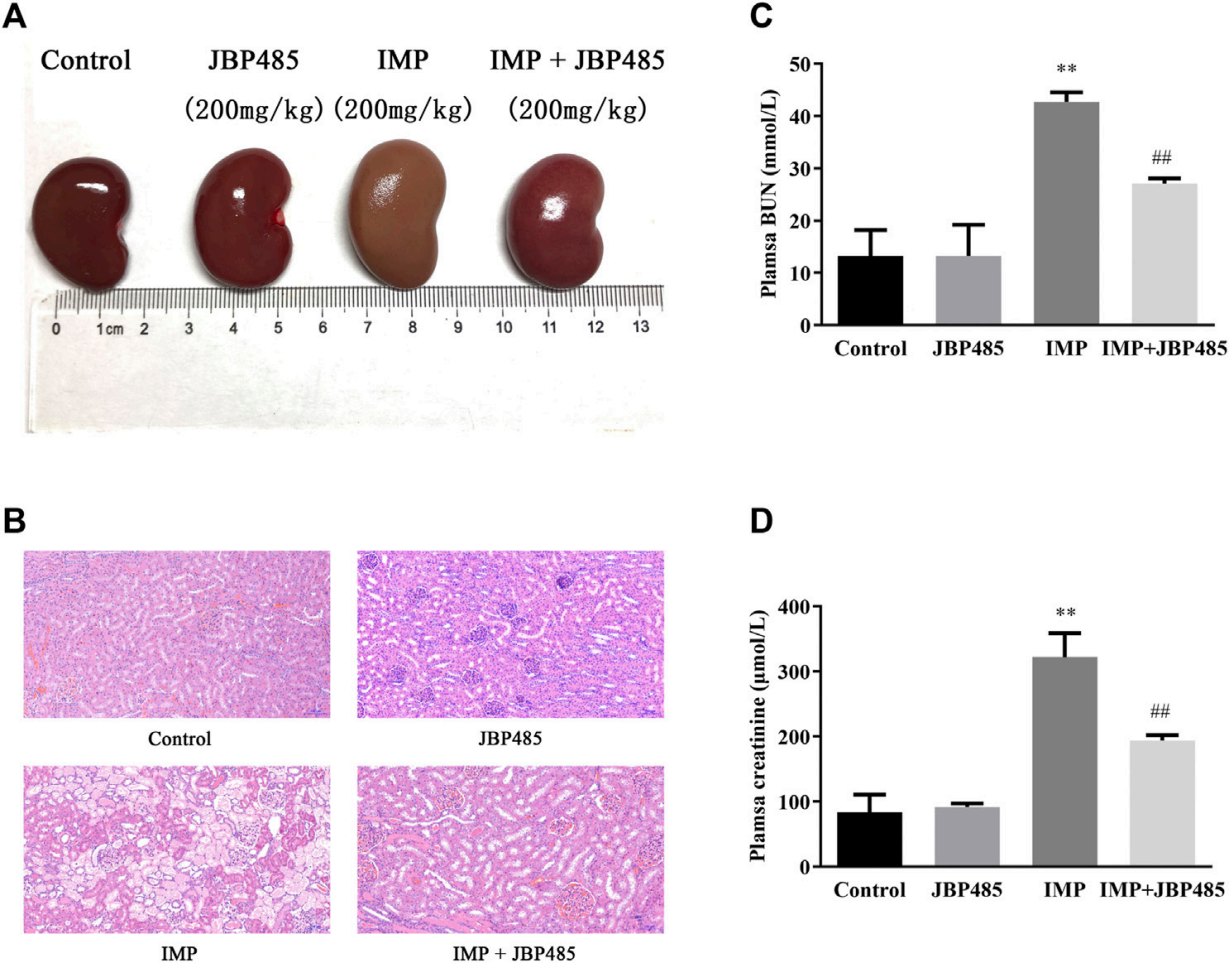
Protective effect of JBP485 on IMP nephrotoxicity in rabbits. Rabbits were injected with IMP (200 mg/kg) through the ear vein in the absence or presence JBP485 (200 mg/kg). The kidneys were collected 72 h after administration of IMP for gross histological observation **(A)** and HE staining **(B)**. Blood samples (0.5 ml) were collected for the determination of BUN **(C)** and CRE **(D)**. Data are expressed as the mean ± SD. **p < 0.01 compared with control; ^##^ *p* < 0.01 compared with IMP group (*n* = 3).

To evaluate the effect of JBP485 on IMP-induced nephrotoxicity in rabbits, IMP was administered to rabbits *via* the ear vein with or without JBP485, and histopathological examinations and renal injury biochemical indicators were used to determine nephrotoxicity. After administration of IMP for 72 h, there was a marked decrease in body weight compared to the control animals. JBP485 alone did not IMP-induced weight loss, although the average body weight was still lower than the control group (Table 1). In contrast to body weight, the kidney weight and the kidney weight to body weight ratio were increased in IMP groups compared with the control group. Coadministration with JBP485 reduced the ratio to the normal level. Furthermore, renal morphology was evaluated by gross histological examinations and HE staining. The kidneys exhibited a normal shape and appearance, aside from those in the IMP group, which were significantly swollen and greyish-yellow in appearance (Figure 1A). Further, histopathological examinations confirmed the protective effect of JBP485 on IMP-induced nephrotoxicity. In the control group, the kidney slices of rabbits exhibited normal renal tissue morphology after HE staining, while the kidney slices of IMP-treated rabbits exhibited serious renal damage characterized, such as decreased glomerular volume, mesangial cell proliferation, and basement membrane thickening, tubular dilation, renal tubular epithelial cell swelling and necrosis (Figure 1B). After coadministration of JBP485, IMP-induced acute structural damage in the rabbit kidneys was significantly reduced (Figure 1B). Moreover, IMP increased the levels of plasma CRE (Figure 1D) and BUN (Figure 1C), and these effects were mitigated by JBP485 treatment. These findings indicate that JBP485 protected against IMP-induced nephrotoxicity.

**TABLE 1:** Effects of JBP485 on IMP-induced changes in body and kidney weights of rabbits.

Group	Body weight (kg)		Δ Weight (g)	Kidney (g)	Kidney/body (g/kg)
	0 h	72 h			
Control	2.55 ± 0.05	2.59 ± 0.05	40.67 ± 1.76	11.37 ± 0 0.29	4.38 ± 0.03
IMP	2.52 ± 0.13	2.37 ± 0.15	−153.33 ± 21.28*	14.20 ± 0.21^*^	6.03 ± 0.30*
JBP485	2.65 ± 0.09	2.69 ± 0.09	41.00 ± 1.53^#^	11.37 ± 0.18^#^	4.23 ± 0.12^#^
IMP + JBP485	2.58 ± 0.05	2.61 ± 0.05	22.67 ± 0.88^*,#^	11.33 ± 0.19^#^	4.35 ± 0.08^#^

Data are expressed as the mean ± SD. *p < 0.05 compared with control; ^#^ *p* < 0.05 compared with IMP group (*n* = 3).

### Protective Effect of JBP485 on IMP-Induced Cytotoxicity in HK-2 Cells

**FIGURE 2:**
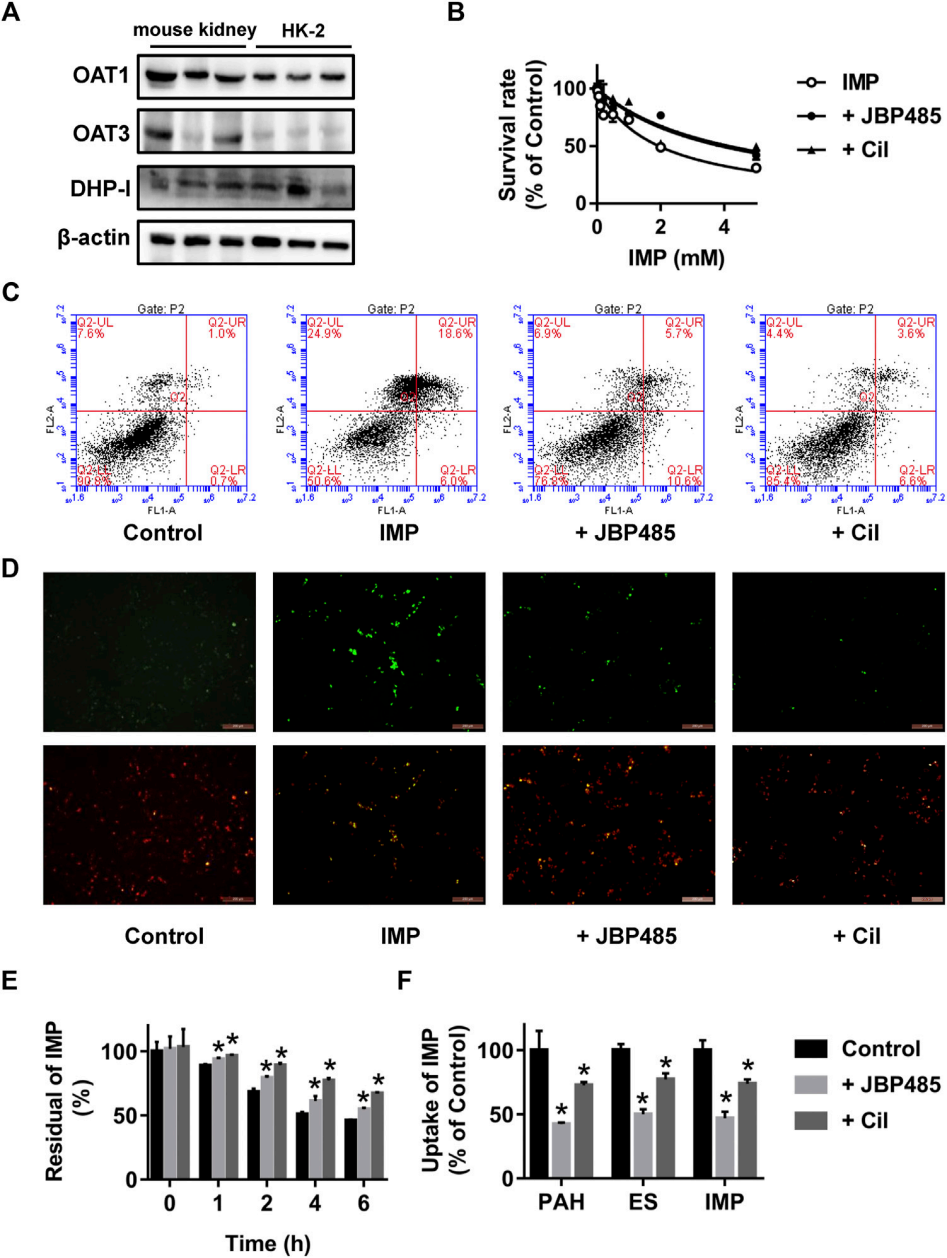
Effect of JBP485 and cilastatin on the cytotoxicity, stability and intracellular accumulation of IMP in HK-2 cells. Protein expression levels of OAT1, OAT3 and DHP-I in HK-2 cells and the mouse kidney by Western blotting **(A)**. HK-2 cells were incubated with IMP (0-5 mM) in the absence or presence of JBP485 (500 μM) or cilastatin (500 μM) for 24 h and cell survival was determined by a CCK-8 assay **(B)**. HK-2 cells were incubated with IMP (2 mM) in the absence or presence of JBP485 (500 μM) or cilastatin (500 μM) for 24 h. Cell apoptosis and the mitochondrial membrane potential were evaluated by Annexin V/PI staining and JC-1 staining assays, respectively **(C,D)**. HK-2 cells were incubated with IMP (50 μM) in the absence or presence of JBP485 (50 μM) or cilastatin (50 μM). The residual concentration of IMP in the medium was determined by LC-MS/MS **(E)**. Intracellular accumulation of IMP in HK-2 cells was determined by LC-MS/MS after incubation with IMP (50 μM) with or without JBP485 (50 μM) or cilastatin (50 μM) for 10 min **(F)**. Data are expressed as the mean ± SD.*p < 0.05 compared with control or IMP group (*n* = 3).

To further clarify the mechanism underlying the protective effect of JBP485 against IMP nephrotoxicity, the cytotoxicity, stability and intracellular accumulation of IMP in HK-2 cells were determined in the presence or absence of JBP485. First, the protein expression levels of OAT1, OAT3 and DHP-I in HK-2 cells were verified by Western blotting. Mouse kidney tissue was used as the positive control. Protein expressions of OAT1, OAT3 and DHP-I were clearly identified in HK-2 cells (Figure 2A), indicating that HK-2 cells could be used to evaluate the interactions mediated by these targets. Then, the effect of JBP485 (500 μM) on IMP-induced cytotoxicity was investigated by determining the cell survival rate, apoptosis and mitochondrial membrane potential. IMP exhibited concentration-dependent cytotoxicity in HK-2 cells, with an IC_50_ value of 1.94 ± 0.19 mM; this value was increased to 4.15 ± 0.62 mM in the presence of JBP485 (Figure 2B). Meanwhile, cell apoptosis and the mitochondrial membrane potential of HK-2 cells were determined through Annexin V/PI staining and JC-1 staining assays after IMP (2 mM) treatment with or without JBP485 (500 μM). The flow cytometry results indicated that JBP485 attenuated IMP-induced apoptosis in HK-2 cells (Figure 2C). Furthermore, IMP induced scattered bright-green fluorescence after JC-1 staining, which was significantly attenuated by co-incubation with JBP485 (Figure 2D). In addition, cilastatin treatment showed a similar protective effect against IMP-induced cytotoxicity (Figures 2B–D). These results suggest that JBP485, as well as cilastatin, protected against IMP-induced cytotoxicity in HK-2 cells. In our previous study (Huo et al., 2019), cilastatin was found to alleviate IMP nephrotoxicity by simultaneously inhibiting renal OATs and DHP-I. Therefore, it was hypothesized that the same mechanism might underlie the effect of JBP485 on IMP nephrotoxicity. Indeed, JBP485 and cilastatin significantly inhibited IMP degradation and improved its stability in the medium of HK-2 cells (Figure 2E). Additionally, intracellular accumulation of IMP, as well as OATs substrates PAH and ES, in HK-2 cells was markedly decreased in the presence of JBP485 or cilastatin (Figure 2F). These findings clearly indicate that JBP485 protected against IMP cytotoxicity in HK-2 cells by improving IMP stability and reducing its intracellular accumulation. Like cilastatin, JBP485 could be a dual inhibitor of renal OATs and DHP-I, which induces a pharmacokinetic DDI and subsequently impacts IMP nephrotoxicity.

### Effect of JBP485 on the Renal Excretion and Plasma Concentration of IMP in Rats

**FIGURE 3:**
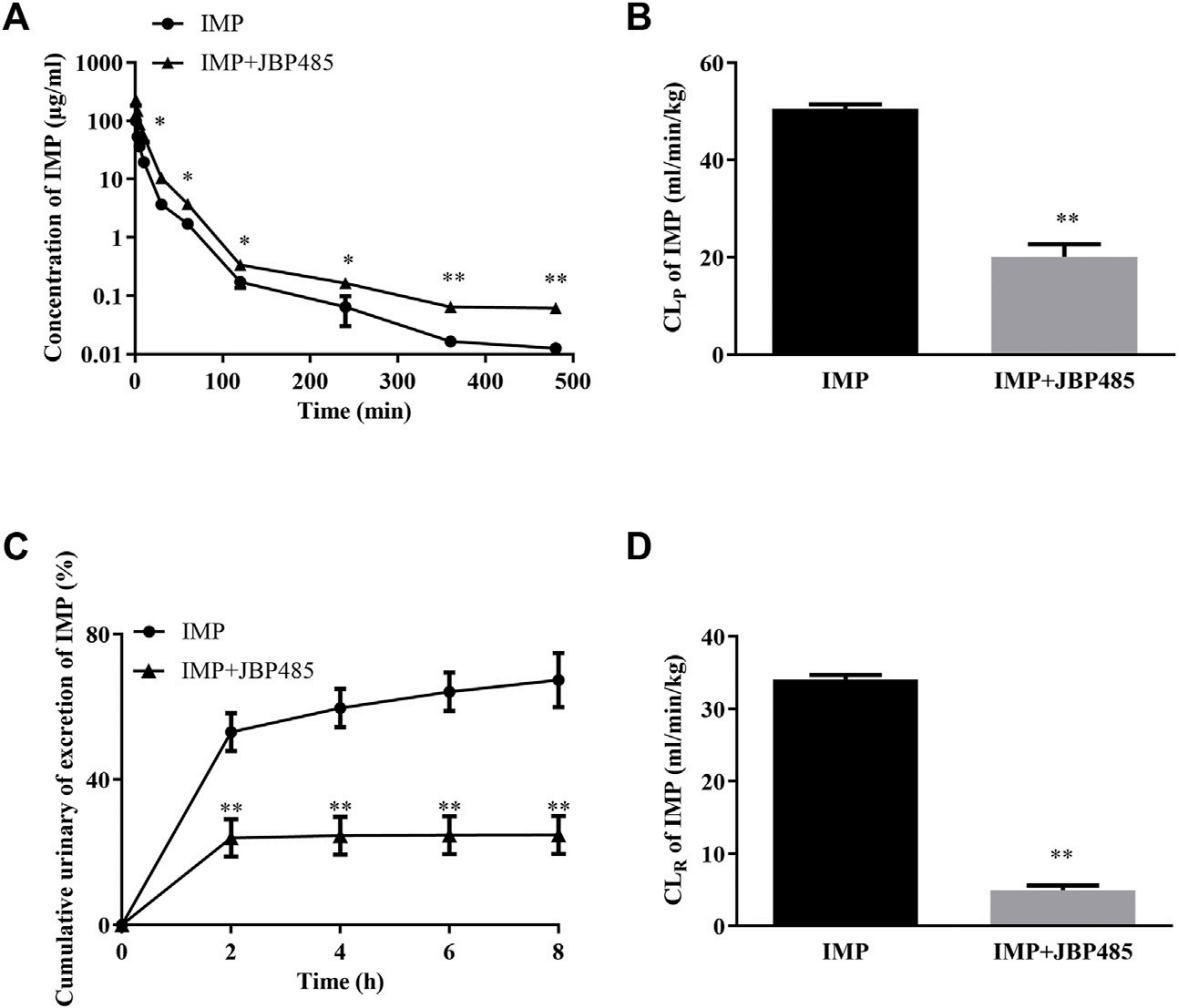
Effect of JBP485 on the pharmacokinetics of IMP in rats. Mean plasma concentration-time curves **(A)**, plasma clearance (CL_P_) **(B)**, cumulative urine excretion curves **(C)**, and renal clearances (CL_R_) **(D)** of IMP after intravenous administration of IMP and JBP485 in rats. Data are expressed as the mean ± SD. *p < 0.05 and **p < 0.01 compared with control (*n* = 5).

To reveal the potential pharmacokinetic DDIs responsible for the protective effect of JBP485 against IMP-induced nephrotoxicity, IMP plasma concentration and cumulative urinary excretion were investigated when IMP and JBP485 were intravenously co-administered. Concurrent administration of JBP485 significantly increased the plasma concentration of IMP. Furthermore, the area under the plasma concentration-time curve (AUC) and half-life (t_1/2β_) of IMP in the coadministration groups were increased (Table 2), while the plasma clearance rate (CL_p_) of IMP was markedly decreased (Table 2; Figures 3A,B). JBP485 significantly decreased cumulative urinary excretions over 8 h and the renal clearance rate (CL_R_) of IMP compared to the IMP alone group (Figures 3C,D). These findings suggest that a pharmacokinetic DDI was induced when IMP and JBP485 were intravenously co-administered and renal excretion of IMP was inhibited by JBP485 in rats.

**TABLE 2:** Pharmacokinetic parameters of IMP after intravenous administration of IMP (45 mg/kg) with or without JBP485 (90 mg/kg) in rats.

Parameter	Unit	IMP	IMP + JBP485
C_0_	μg/ml	137.7 ± 6.1	279.3 ± 6.3**
AUC_0-∞_	μg/ml·min	890.8 ± 16.8	2,267.7 ± 309.6*
t_1/2β_	h	1.2 ± 0.4	2.3 ± 0.1*
CL_p_	ml/min/kg	50.5 ± 1.0	20.1 ± 2.6**
CL_R_	ml/min/kg	34.0 ± 0.6	5.0 ± 0.6**

Data are expressed as the mean ± SD. ∗*p* < 0.05 and ∗∗*p* < 0.01 compared with IMP group (*n* = 3).

### Effect of JBP485 and Cilastatin on IMP Uptake in Kidney Slices and hOAT1-/hOAT3-Transfected HEK293 Cells

**FIGURE 4:**
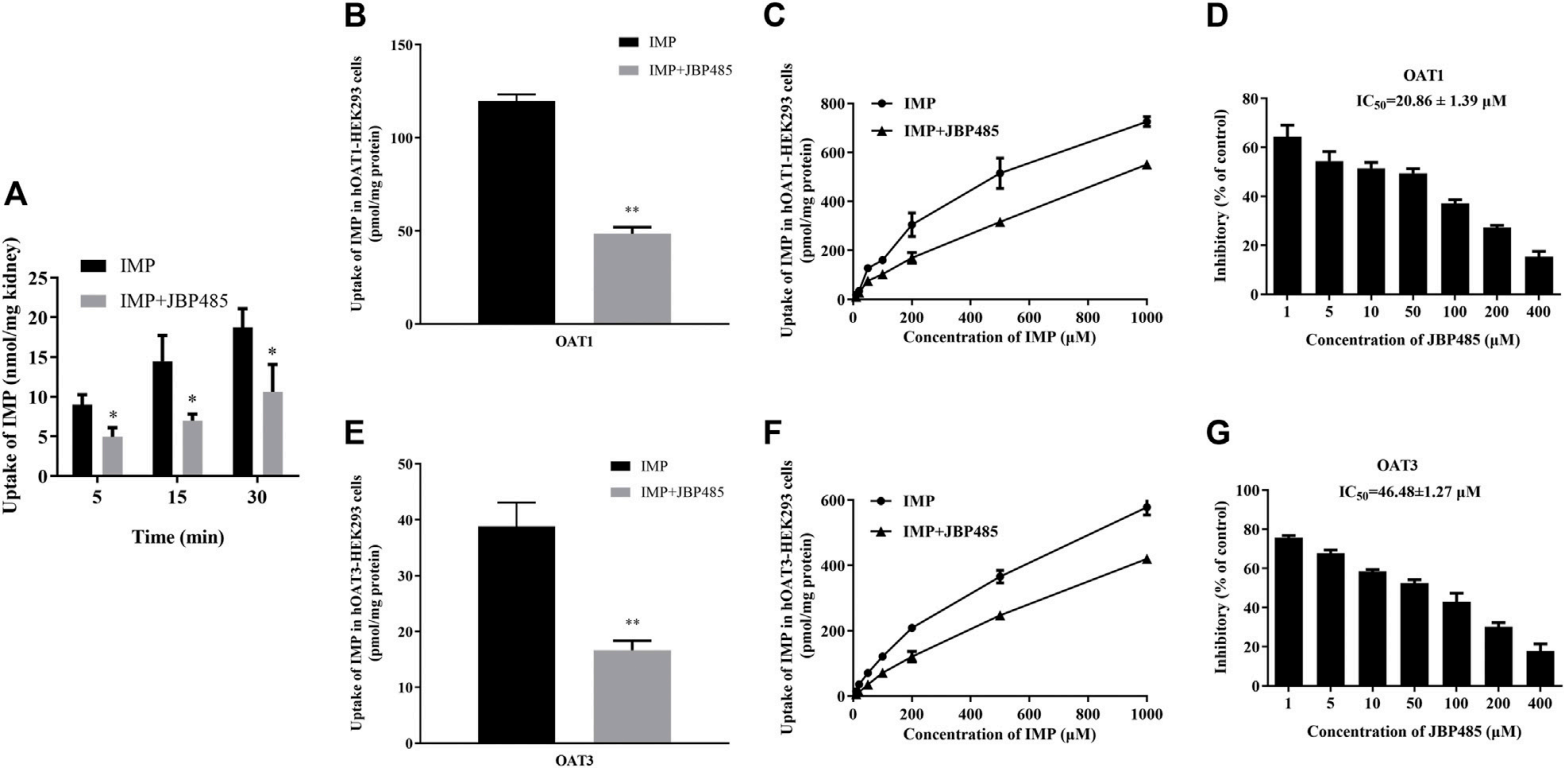
Effect of JBP485 on the uptake of IMP by rat kidney slices and hOAT1/3-HEK293 cells. Inhibition effect of JBP485 (50 μM) on the uptake of IMP (50 μM) in kidney slices **(A)**. Inhibition effect of JBP485 (50 μM) on the uptake of IMP (50 μM) in hOAT1/3-HEK293 cells **(B,E)**. Inhibition effect of JBP485 (50 μM) on the uptake of IMP (10–1,000 μM) in hOAT1/3-HEK293 cells **(C,F)**. Inhibition effect of JBP485 (1–400 μM) on the uptake of IMP (50 μM) in hOAT1/3-HEK293 cells **(D,G)**. Data are expressed as the mean ± SD. *p < 0.05 and ** *p* < 0.01 compared with control (*n* = 3).

To exclude the influence of physiologic conditions, fresh rat kidney slices and hOAT1-/hOAT3-transfected HEK293 cells were used to investigate the target transporters involved in the DDI between IMP and JBP485. First, JBP485 was found to significantly inhibit IMP uptake in a time-dependent manner (Figure 4A), suggesting that the DDIs between IMP and JBP485 occurred in the kidney and JBP485 inhibited the renal distribution of IMP. Meanwhile, the effects of JBP485 on the uptake of IMP in hOAT1- and hOAT3-HEK293 cells were verified. The uptake of IMP in hOAT1- and hOAT3-HEK293 cells was inhibited for 10 min following the addition of JBP485 (Figures 4B,E). Intracellular levels of IMP in hOAT1- and hOAT3-HEK293 cells were decreased by JBP485 in a concentration-dependent manner, with IC_50_ values of 20.86 ± 1.39 μM (for OAT1) and 46.48 ± 1.27 μM (for OAT3) (Figures 4D,G). Moreover, JBP485 inhibited uptake of IMP at varying concentrations in hOAT1- and hOAT3- HEK293 cells (Figures 4C,F). Eadie-Hofstee plot analysis showed that JBP485 significantly increased the K_m_ values of IMP in hOAT1- and hOAT3-HEK293 cells, but did not change V_max_ values (Table 3), suggesting competitive inhibition. These results confirm that renal OATs were at least one target of the DDI between JBP485 and IMP.

**TABLE 3:** K_m_ and V_max_ values of IMP with or without JBP485 in hOAT1-HEK293 cells and hOAT3-HEK293 cells.

Group	hOAT1-HEK293 cells		hOAT3-HEK293 cells	
	K_m_	V_max_	K_m_	V_max_
**IMP**	0.564 ± 0.048	0.300 ± 0.027	0.581 ± 0.064	0.450 ± 0.038
**IMP + JBP485**	0.878 ± 0.024^*^	0.285 ± 0.021	0.994 ± 0.024^*^	0.438 ± 0.021

Data are expressed as the mean ± SD. ^∗^ *p* < 0.05 compared with IMP group (*n* = 3).

### Effect of JBP485 on IMP Metabolism in Rat Kidney

**FIGURE 5:**
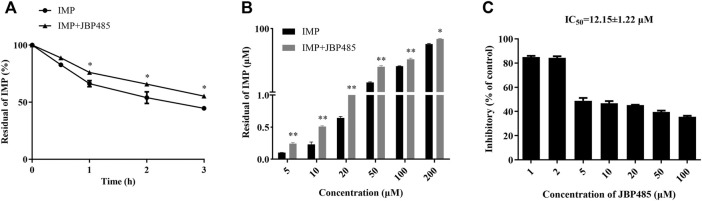
Effects of JBP485 on IMP metabolism in rat kidney. Metabolism of IMP (50 μM) by rat kidney in the presence or absence of JBP485 (50 μM) at 0.5, 1, 2 and 3 h **(A)**. Concentration-dependant metabolism of IMP (5–200 μM) with or without JBP485 (50 μM) **(B)**. Inhibitory effect of JBP485 (1–100 μM) on IMP (50 μM) metabolism in rat kidney **(C)**. Data are expressed as the mean ± SD. * *p* < 0.05 and ** *p* < 0.01 compared with control (*n* = 3).

Previous studies have demonstrated that OATs and DHP-I may be involved in the protective effect of cilastatin on IMP-induced kidney injury and that the metabolites of IMP might have more potent cytotoxicity than IMP (Huo et al., 2019). Current results indicated that JBP485 inhibited IMP renal excretion by OATs. Thus, we further examined whether JBP485 affected the metabolism of IMP. The residual of IMP at different times and concentrations were significantly increased in the presence of JBP485 (Figures 5A,B). The IC_50_ value of JBP485 for IMP metabolism by renal DHP-I was calculated to be 12.15 ± 1.22 μM (Figure 5C). These findings confirm that JBP485 improved the stability of IMP in the rat kidney, which consequently reduced the exposure to toxic metabolites of IMP.

### Molecular Docking Simulation

**FIGURE 6:**
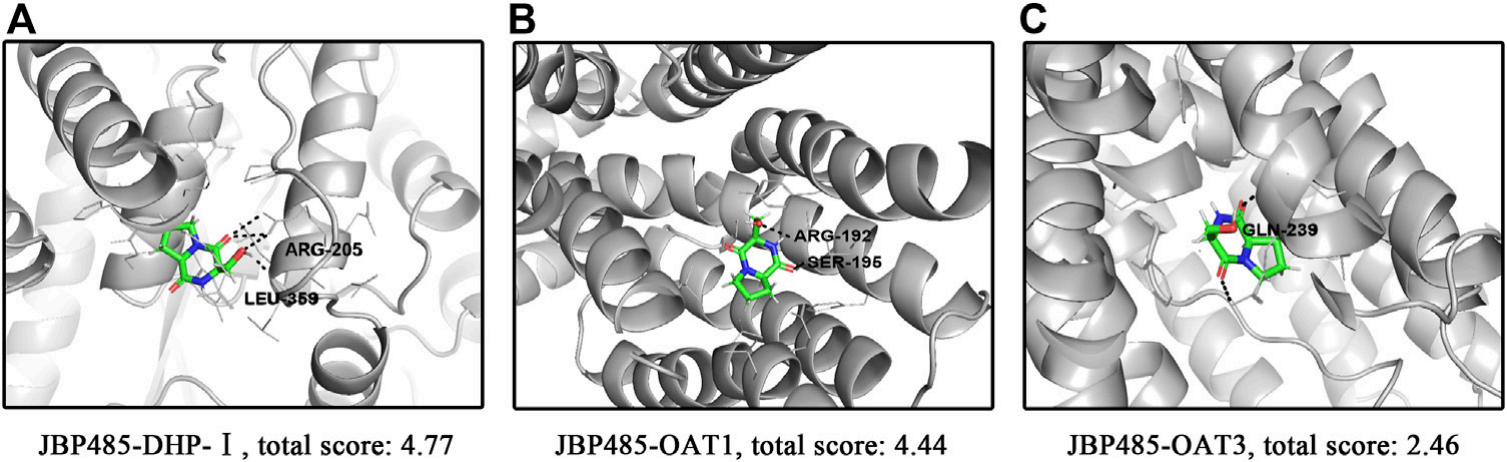
Molecular docking simulation between JBP485 and DHP-I **(A)**, OAT1 **(B)** or OAT3 **(C)**.

Molecular docking was conducted to explore the molecular interactions between JBP485 and DHP-I, OAT1 and OAT3 (Figure 6). The optimal confirmation and total score of JBP485 within these three proteins were compared. As shown in Figure 6, JBP485 formed two H-bonds with ARG-205 and LEU-359 of DHP-I, yielding a total score of 4.77, suggesting a good affinity of JBP485 with DHP-1. Further, two H-bonds were formed between JBP485 and OAT1 (ARG-192 and SER-195) with a total score of 4.44. However, only one H-bond was found between JBP485 and OAT3 (with GLN-239), with a total score of 2.46. This suggests that JBP485 exhibited a higher affinity to OAT1 than OAT3.

## Discussion

IMP is a carbapenem antibiotic with potent antibacterial activity. However, when IMP is administered alone, it is quickly degraded by DHP-I. DHP-I is a membrane enzyme expressed in the kidneys that hydrolyses a variety of dipeptides. To improve IMP stability, cilastatin, as a potent DHP-I inhibitor, was developed and was found to successfully increase IMP urinary recovery. Our previous studies demonstrated that IMP and cilastatin are substrates of hOAT1 and hOAT3 (Huo et al., 2019; Zhu et al., 2020). Cilastatin was found to inhibit IMP uptake by OATs and reduce renal exposure to IMP, which consequently protected against kidney injury induced by IMP *in vitro* and *in vivo*. Both OATs and DHP-I were found to play vital roles in the renal disposition of IMP and thus, could serve as potential therapeutic targets to prevent IMP nephrotoxicity. JBP485 is a substrate of OATs (Zhang et al., 2010) and exhibits protection against gentamicin or cisplatin-induced renal injury in rats (Liu et al., 2012; Guo et al., 2013). Thus, the present study investigated whether JBP485 could relieve the kidney injury induced by IMP and explored the underlying mechanism.

### JBP485 Attenuated IMP Nephrotoxicity *In Vivo* and *In Vitro* Through a Pharmacokinetic DDI

Because rabbits are susceptible to IMP nephrotoxicity (Birnbaum et al., 1985), rabbits were used to evaluate the nephrotoxicity of IMP and the protective effect of JBP485. Nephrotoxicity of IMP was examined after the intravenous administrations of IMP (200 mg/kg) to rabbit (Lim et al., 2008; Huo et al., 2019; Huo et al., 2020; Lyon, 1985). Coadministration of cilastatin (200 mg/kg) prevented the changes induced by Imp in rabbits (Huo et al., 2019). We selected equal dosage of JBP485 (200 mg/kg) to investigate its preventive and therapeutic effect on IMP-induced kidney injury. In JBP485 alone group, BUN, CRE and the shape and appearance of the kidney were similar with control group. This indicated that JBP485 at 200 mg/kg had no toxic effects on rabbit kidneys. Co-administration of IMP with JBP485 significantly improved the renal dysfunction and pathological changes induced by IMP (Figure 1 and Table 1). This suggests that JBP485 protected against IMP-induced renal injury in rabbits. IMP nephrotoxicity has been characterized as mitochondrial damage and renal tubular cell apoptosis (Huo et al., 2019; Cherif et al., 2019). In order to further clarify the protective effect of JBP485 against IMP-induced kidney injury, HK-2 cells were used to avoid the influence of changes in physiological conditions and to predict the effects in humans. HK-2 cells are human-derived proximal tubular epithelial cells and have been commonly used as an *in vitro* model to evaluate drug nephrotoxicity and the potential mechanisms of renal diseases (Qiu et al., 2018; Bejoy et al., 2022). The uremic toxins indoxyl sulphate and p-Cresyl sulphate are taken up into HK-2 cells via OATs and induce cytotoxicity in HK-2 cells, suggesting that OATs are expressed in HK-2 cells and contribute to the cytotoxicity of toxins (Motojima et al., 2003; Miyamoto et al., 2011; Nakano et al., 2021). In the present study, protein expressions of both OAT1/3 and DHP-I were identified in HK-2 cells, which confirms that HK-2 cells are a suitable model for the investigation of DDIs mediated by OAT1/3 and DHP-I. Incubation with JBP485 significantly ameliorated IMP-induced cell apoptosis and mitochondrial dysfunction (Figures 2B–D). This confirms the protective effect of JBP485. Meanwhile, JBP485 improved IMP stability and reduced the intracellular accumulation of IMP in HK-2 cells (Figures 2E,F). Importantly, these effects of JBP485 were similar to those of cilastatin (Figures 2B–F), suggesting that JBP485 is an effective substitute for cilastatin and could serve as a promising agent to reduce the adverse renal effects of IMP in the clinical setting. In our previous studies, IMP metabolites were found to exhibit higher toxicity than the parent drug, and cilastatin ameliorated IMP nephrotoxicity both in rabbits and *in vitro* through inhibition of both OATs and DHP-I (Zhu et al., 2020). In a previous study, we found that the natural product Apigenin protects against IMP nephrotoxicity through OAT inhibition (Mei et al., 2020). Despite its potent inhibitory capacity against OATs, Apigenin did not improve the stability of IMP (Huo et al., 2020). In the present study, JBP485 attenuated IMP nephrotoxicity, improved its stability, and decreased intracellular exposure, which suggests that JBP485 might serve as a promising dual inhibitor of OATs and DHP-I, and can attenuate IMP nephrotoxicity through a pharmacokinetic DDI.

### JBP485 Inhibited Renal OATs and DHP-I

To further investigate the target of the DDI between IMP and JBP485, we focused on OATs which are important transporters in the kidney. Renal transport mediated by OATs facilitates substrate uptake into the kidney, which, conversely leads to higher exposure and then increases the toxic risk (Wang and Sweet, 2013; Nigam, 2015; Huo and Liu, 2018). IMP at 45 mg/kg was used in pharmacokinetic study in rats according to therapeutic doses of IMP commonly used clinically and in previous research (Zhu et al., 2020). The therapeutic dose of IMP or cilastatin commonly used in clinic is 500 mg iv (equal to 7.14 mg/kg), which converting to dose in rats is about 45 mg/kg. The 6.3 fold of human dose equals to the dose of rats, which is a simple method for converting the dosage from human to rats. JBP485 was then administered in a 1:1 and 1:2 ratio with IMP (45 mg/kg). The inhibition of JBP485 (90 mg/kg) on IMP renal elimination was more pronounced than JBP485 (45 mg/kg). In our previous study, JBP485 (100 mg/kg) safely and effectively relived acute renal failure (ARF) induced by cisplatin (Liu et al., 2012). Therefore, we selected the dose of 90 mg/kg to elucidate the effect of JBP485 on renal clearance of IMP in rats. In the present study, urinary secretion of IMP was investigated in rats via bladder intubation, considering the need for rapid sampling to maintain IMP stability. The results revealed that the rats that received the combination of IMP and JBP485 exhibited higher plasma concentrations of IMP, lower cumulative urinary excretion of IMP, and a lower CL_R_ (Table 2; Figure 3). These findings indicate that JBP485 inhibited the renal elimination of IMP. Numerous studies have shown that JBP485 can inhibit the renal excretion of OAT substrates, such as bestatin (Zhu et al., 2012), acyclovir (Ye et al., 2012) and entecavir (Xu et al., 2013). Thus, it was hypothesized that OATs might be involved in the DDI between IMP and JBP485. To test this hypothesis, the effects of JBP485 on IMP uptake in kidney slices and hOAT1/3-HEK293 cells were investigated. The uptake of IMP in both kidney slices and transfected cells decreased in the presence of JBP485 (Figure 4). Kinetic analysis confirmed that JBP485 significantly increased the K_m_ values of IMP in hOAT1- and hOAT3-HEK293 cells, but did not change V_max_ values (Table 3). This indicates that JBP485, as a substrate of hOAT1 and hOAT3, inhibited the uptake of IMP by hOAT1/3 in a competitive way. Together, these results indicate that hOAT1 and hOAT3 are the target transporters involved in the DDIs between IMP and JBP485 in the kidney.

After IMP enters the kidney, it was rapidly catalysed to inactive and toxic metabolites by DHP-I. This phenomenon not just decreases the activity and effectiveness of IMP, but also can result in kidney injury (Hakeam et al., 2019). In previous research, when cilastatin binds with DHP-I it can interact with apical cholesterol lipid rafts and, therefore, fight off apoptosis and oxidative stress caused by nephrotoxic medications (Shayan and Elyasi, 2020). In addition, cilastatin inhibits DHP-I and successfully prevents the renal damage induced by IMP. Further, cilastatin exerts promising protective effects against a number of other nephrotoxic agents such as vancomycin (Im et al., 2017), diclofenac and cisplatin (Camano et al., 2010; Moreno-Gordaliza et al., 2011; Humanes et al., 2012; Humanes et al., 2017; Moreno-Gordaliza et al., 2018). JBP485 has been confirmed to protect against vancomycin- or cisplatin-induced ARF in rats (Liu et al., 2012; Wen et al., 2018). In the present study, JBP485 was found to inhibit IMP uptake by hOAT1/3, decrease hOAT1/3-dependent cytotoxicity and relieve IMP-induced renal injury. However, to date, there are no studies examining whether JBP485 can inhibit the metabolism of IMP by DHP-I. Thus, a kidney metabolism experiment was performed to investigate the effect of JBP485 on IMP metabolism in the kidneys. The results indicated that JBP485 can inhibit the metabolism of IMP (Figure 5). In summary, the protective effect of JBP485 against IMP-induced renal injury was partly due to the inhibition of IMP metabolism by DHP-I.

It has been demonstrated that JBP485 can inhibit OATs transport as well as DHP-I metabolism of IMP. The molecular docking results also confirmed the affinities of JBP485 to DHP-I, OAT1 and OAT3. Previous studies have shown that IMP has minimal affinity for OATs (Suzuki et al., 1989). Thus, we surmised that the transport of OATs was the rate-limiting step in IMP renal elimination. The *in vivo* results demonstrated that when JBP485 was co-administered with IMP, IMP cumulative urinary excretion was significantly reduced. JBP485 had a greater contribution to inhibiting OAT1/3-mediated transport, thus decreasing the renal uptake of IMP. Meanwhile, JBP485 inhibited the metabolism of IMP (part of which is taken up by the kidneys) to toxic metabolites. These two different mechanisms can both attenuate IMP-induced nephrotoxicity.

JBP485 isolated from Laennec can be synthesized by chemical means and is completely free from any pathogens (Nagata et al., 2015). Laennec has been clinically used to treat chronic hepatic injuries for over forty years in Japan (Liu et al., 2000). In systemic administration, JBP485 has already been shown to have no adverse effects in rats and mice (Liu et al., 2000). JBP485 is not toxic for the kidney and in fact protects against renal toxicity as the substrate of OATs. In summary, there were no relevant research about the toxicity and side effects caused by co-administration of JBP485. In present study, JBP485 was well tolerated in rabbits (200 mg/kg) and rats (90 mg/kg). Meanwhile, JBP485 effectively protected the kidney against IMP-induced renal toxicity, suggesting its well established safety and efficacy in animals *in vivo*. On the other hand, the IC_50_ values of JBP485 on DHP-I and OATs were approximate 12 μM and 20-50 μM (Figure 4 and Figure 5). According to FDA guideline, clinical DDI may occur when [I]/IC_50_ is higher than 0.1, suggesting that human plasma concentrations of JBP485 higher than 1-5 μM could induce significant DDI between JBP485 and IMP in human. However, the plasma concentrations of JBP485 in human was not available. In our previous study, C_max_ of JBP485 in rats after intravenous administration of JBP485 (25 mg/kg) was higher than 650 μM (Zhang et al., 2010), which was high enough to inhibit DHP-I and OATs *in vivo*. Therefore, JBP485 (500 μM) was used to evaluate its protective effect in HK-2 cells. Overall, the dosage or concentration of JBP485 used in present study was safe, effective, and achievable in human. Consequently, the findings have clinical significance.

In conclusion, JBP485 relieved IMP-induced renal injury *in vivo* and *in vitro*. OATs and DHP-I mediated the DDI between IMP and JBP485, which reduced renal exposure to IMP and its toxic metabolites. Thus, JBP485 can serve as a promising agent to reduce the adverse renal effects of IMP in the clinical setting.

## Data Availability

The original contributions presented in the study are included in the article/Supplementary Material, further inquiries can be directed to the corresponding authors.

## Abbreviations

AUC, area under the plasma concentration-time curve; BUN, blood urea nitrogen; CRE, creatinine; CLp, plasma clearance; CLR, renal clearance; DHP-I, renal dehydropeptidase-I; DDIs, drug-drug interactions; hOAT, human OAT; HEK293, human embryonic kidney 293; HK-2, human kidney 2; IMP, imipenem; LC-MS/MS, liquid chromatography–tandem mass spectrometry; OATs, renal organic anion transporters; PAH, p-aminophenol acid; t1/2β, half-life.
